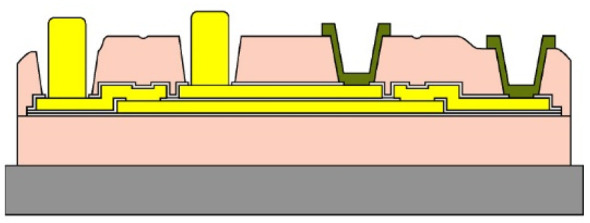# Micro-Fabrication of Components for a High-Density Sub-Retinal Visual Prosthesis

**DOI:** 10.3390/mi11100944

**Published:** 2020-10-19

**Authors:** Douglas B. Shire, Marcus D. Gingerich, Patricia I. Wong, Michael Skvarla, Stuart F. Cogan, Jinghua Chen, Wei Wang, Joseph F. Rizzo

**Affiliations:** 1 Bionic Eye Technologies, Inc., Ithaca, NY 14850, USA; mdg37@cornell.edu (M.D.G.); pwong@bionicvisiontechnologies.com (P.I.W.); 2 Cornell NanoScale Science and Technology Facility, Ithaca, NY 14853, USA; skvarla@cnf.cornell.edu; 3 Department of Bioengineering, University of Texas, Dallas, Richardson, TX 75080, USA; sxc149830@utdallas.edu; 4 Department of Ophthalmology, University of Florida, Gainesville, FL 32611, USA; jinghuachen@ufl.edu; 5 Department of Ophthalmology, University of Louisville, Louisville, KY 40292, USA; wei.wang@louisville.edu; 6 Massachusetts Eye and Ear, Boston, MA 02114, USA; joseph_rizzo@meei.harvard.edu

**Keywords:** microfabrication, visual, prosthesis, vision, electrode, neurostimulator, wireless, high-density, retina, neurostimulation

## Abstract

We present a retrospective of unique micro-fabrication problems and solutions that were encountered through over 10 years of retinal prosthesis product development, first for the Boston Retinal Implant Project initiated at the Massachusetts Institute of Technology and at Harvard Medical School’s teaching hospital, the Massachusetts Eye and Ear—and later at the startup company Bionic Eye Technologies, by some of the same personnel. These efforts culminated in the fabrication and assembly of 256+ channel visual prosthesis devices having flexible multi-electrode arrays that were successfully implanted sub-retinally in mini-pig animal models as part of our pre-clinical testing program. We report on the processing of the flexible multi-layered, planar and penetrating high-density electrode arrays, surgical tools for sub-retinal implantation, and other parts such as coil supports that facilitated the implantation of the peri-ocular device components. We begin with an overview of the implantable portion of our visual prosthesis system design, and describe in detail the micro-fabrication methods for creating the parts of our system that were assembled outside of our hermetically-sealed electronics package. We also note the unique surgical challenges that sub-retinal implantation of our micro-fabricated components presented, and how some of those issues were addressed through design, materials selection, and fabrication approaches.

## 1. Introduction

The retinal neural network is a part of the brain with well-understood anatomical and physiological organization. Patients with longstanding and profound blindness secondary to retinal disease have provided detailed feedback on perceptual responses to electrical stimulation of the retina, whether the stimuli were provided sub-retinally or epi-retinally [1,2,3,4]. Advances in micro-fabrication technology, electronics, materials, surgical techniques, and other research have enabled our Bionic Eye Technologies team (formerly known as the Boston Retinal Implant Project or BRIP) to build a 256+ channel retinal prosthesis to restore vision to patients who have lost vision due to degenerative diseases such as retinitis pigmentosa and age-related macular degeneration [5]. In recent years, the visual prosthetic community of researchers and commercial entities has expanded to well over 20 groups worldwide [4,6,7,8,9,10,11,12,13,14]. Remarkable strides have been made toward the restoration of useful vision to severely blind patients by several teams, including recent promising results by the Pixium company in subjects with macular degeneration [15]. Collectively, the results to date are encouraging, but the field as a whole falls shy of the goal of creating natural, high quality vision that could justify the risks of surgery and the high cost of the devices. These issues among others have led in part to modest market penetration by existing prosthetic devices that have obtained regulatory approval, and even to the closure of some companies that produced them. The need to achieve higher quality of restored vision has motivated us, as well as other teams, to develop more advanced, high-density devices and to seek to introduce stimuli to the target tissue with closer apposition of the stimulating electrodes. In this regard, the approach of our team has been to further develop penetrating sub-retinal electrodes as we prepared devices for pre-clinical testing, and we are now striving to demonstrate the potential advantages of such a design over the planar electrodes that have been a standard for use by many groups. 

The focus of this report is on the micro-fabrication methods that have been used to create the implantable components of our sub-retinal prostheses that lie outside of the electronics package. We address in detail the creation of an electrode array that is introduced into the sub-retinal space using an *ab externo* (“from the outside”) surgical approach, and the means to facilitate the implantation of these flexible arrays through a flap created in the sclera. More recently, this effort has included the addition of flexible structures that incorporate penetrating electrodes, which we have successfully implanted using the same techniques as the planar arrays [16,17]. We also discuss the processing and assembly of supporting micro-fabricated components that facilitated the surgical placement of the device components around the exterior of the eye, including the “secondary” radio frequency coil that provides power bi-directional wireless data (e.g., digitized details of the visual scene) to and from the “primary” radio frequency coil that is embedded within a pair of glasses worn by the user. Our micro-fabrication and surgical methods have been proven in extensive pre-clinical tests in miniature pig animal models. We are cautiously hopeful that our penetrating electrodes, which will deliver stimulation to small clusters of nearby neurons, will improve the quality of vision over what can be obtained with planar surface electrodes.

Early in the development of prior generations of our visual prosthesis, we elected to build a device that would introduce electrical stimuli at a sub-retinal location, that is, adjacent to the zone where photoreceptor cells are lost in the degenerative diseases that we were targeting. This was a fundamental strategic change from the decade long experience we had working on the epiretinal surface and even performing acute (i.e., short duration) proof-of-concept retinal stimulation tests on blind volunteers [1,2]. In essence, we accepted the consequences of a “trade-off” in that the surgical approach to the sub-retinal space requires breaching the highly vascular choroid, and the technical requirements were more challenging compared to conventional vitreo-retinal surgery. Nonetheless, we gradually became convinced that the engineering and biological advantages of a sub-retinal approach would more likely lead to improved clinical results, as has been the case in recent studies by others [15]. We believe that these results may improve further by introducing highly localized electrical stimuli atop and/or beside penetrating posts that support the electrode structures. Indeed, we have fabricated such high-density 3D penetrating electrode arrays for our own devices, as have other groups more recently [18,19,20,21], and we report on their fabrication technology below. 

Micro-fabrication is one of several key technological drivers that together enable higher channel counts for implantable neuro-stimulators generally, and for vision prostheses in particular. Unlike cochlear implants for the deaf, which can produce meaningful auditory percepts using modest numbers of electrodes, visual prostheses can require hundreds of individually-controllable stimulation channels to induce reasonably high-density phosphenes so that detailed images can be interpreted [8,22]. There are several micro-fabrication challenges to creating high-density flexible electrode arrays suitable for the sub-retinal space, and they are summarized below in Table 1. The aim of this work was to address these challenges in an integrated, multi-disciplinary manner in consultation with our surgical team members, and to produce robust, multi-layered, flexible components for our visual prostheses that could survive the implantation procedure and last in the body for perhaps up to 10 years thereafter. In reporting our results here, we emphasize the fabrication-related challenges that we faced which were more universal in nature, as opposed to those related solely to any technical limitations of the micro-processing equipment that we had at our disposal.

## 2. Materials and Methods 

**Figure 1:**
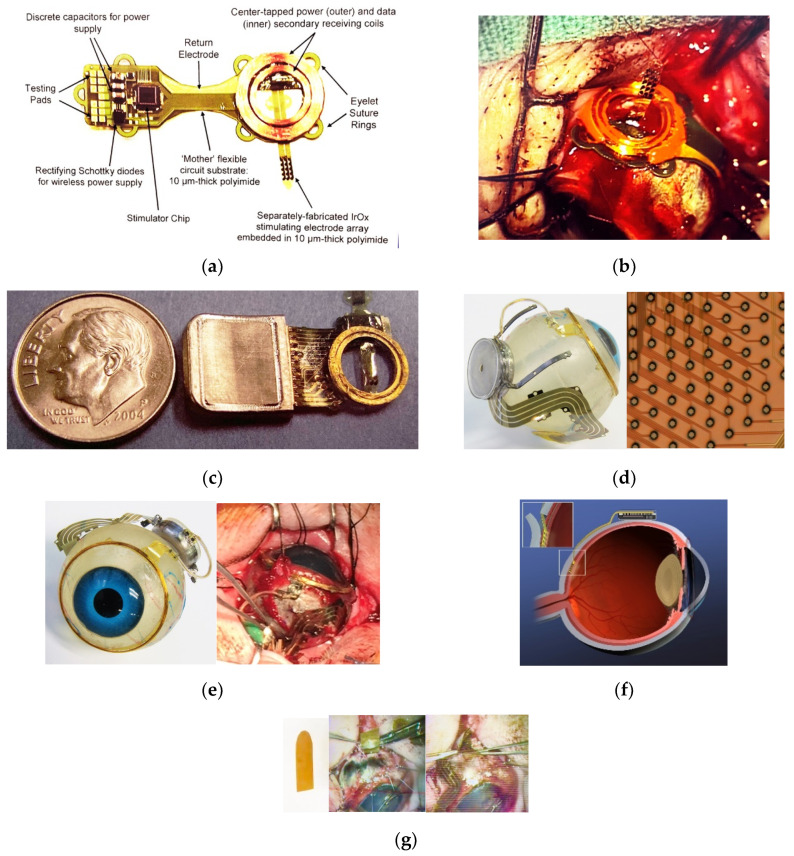
Three generations of the Boston Retinal Prosthesis with micro-fabricated components. (**a**,**b**) First generation 16-channel stimulator with early micro-fabricated electrode array in mini-pig model; (**c**) second-generation hermetically packaged 16-channel device with bidirectional telemetry; (**d**,**e**) third-generation hermetically-packaged 256+ channel unit in minipig, and bonded array-feedthrough interconnections prior to final silicone overmolding—the electrode array is 5 mm at widest with segmented leads, and the package is 11 mm in diameter; (**f**) a simplified cross-sectional cartoon of the implant showing the package mounting on the eyeball, the leads along the outside of the eye traversing the eye wall into the subretinal space (inset); (**g**) a 5 mm wide, 15 mm long and 75 μm thick polyimide surgical guide, and the guide in use during array insertion.

The fabrication related challenges that our team addressed in building implantable visual prostheses are outlined below. We have constructed three generations of retinal implants, and the implantable components can be seen in Figure 1. The complete visual prosthesis system contains an external controller with a radio frequency transceiver, but that system does not make use of any unique micro-fabrication technology, and is not reported on here. Likewise, the application-specific integrated circuits (ASICs) for providing charge-balanced stimuli to retinal tissue were sourced from established mixed-signal chip foundry services, and have been described elsewhere [23]. The ASICs were mounted on internal, high-density multilayer flexible circuit boards using flip-chip/ surface-mount assembly methods that are well established in the industry. Our approach was to attach the multi-layered, flexible internal circuit board to the interior surface of a high temperature, co-fired (HTCC) alumina ceramic feedthrough that formed the lid of our round hermetic package (see Figure 1). The package materials (alumina, ceramic, and platinum feedthroughs) and our gold brazing methods are in common use in the implantable medical device industry, and are outlined in [24]. Here, we will focus on the flexible multi-electrode arrays that deliver stimuli from the implanted, sealed wirelessly powered stimulator units to the retina, and the microfabricated supporting structures for the secondary radio frequency coils.

### 2.1. Mechanical Properties and Design Considerations for Retinal Prostheses

Each fabrication and surgical challenge that we encountered along the way influenced not only the CAD design of the components and their multilayered construction, but also the order of fabrication and the choices of materials for processing. As well, we obtained practical feedback on both the layout and the stiffness and handling of the components by performing many pre-clinical animal surgical procedures in miniature pig models, and these too informed our design choices; see e.g., [16]. Further feedback came from in vitro testing, particularly accelerated lifetime testing in biological saline, which influenced our material selection. The design elements and challenges that we addressed also interacted with one another; the mechanical design problems facing the creation of micro-fabricated implantable components for a sub-retinal visual prosthesis are, in fact, multi-faceted. For example, our multi-electrode arrays for retinal stimulation must cross the sclera and the choroid for their leads to convey signals from a peri-ocular, hermetically packaged implanted stimulator unit to the distal electrodes in the sub-retinal space. The peri-ocular portion of our implantable visual prosthesis is subject to different mechanical stresses during and after the surgical procedure than the intra-ocular portion. For instance, the extra-ocular portion may rub against and possibly erode overlying tissue such as the conjunctiva and Tenon’s capsule, which we observed early in the development of our second-generation device [25]. This outcome resulted in modifications to the most anterior part of the peri-ocular implant to reduce its profile as much as possible. For example, the 19 mm diameter secondary radio frequency coil, which in our case is implanted and sutured around the cornea, was at one time encapsulated in a ~2 mm thick layer of polydimethylsiloxane (PDMS). This gave rise to undue tension on the overlying conjunctiva at the end of the procedure. This recurrent outcome led to an effort to micro-fabricate the coils using a SU-8 epoxy mold and gold electroplating techniques. However, ultimately we found that the best solution to mechanical stresses on the RF coil and the surrounding tissue was to wind the coils from 75 μm diameter gold wire on a spherical form of the same diameter as the target eye. By careful attention to the layering of the turns, we were able to reduce the height of the coil assembly to <0.5 mm, which provided long-term biocompatibility. We later elected in our third-generation device to microfabricate a coil support structure from electroplated Au and polyimide that provided attachment points for suturing the coil to the sclera. Even this simple structure, shown assembled in Figure 1 and discussed further below, had mechanical design considerations that were resolved through trial-and-error in multiple implantations in mini-pig animal models. For example, eye size impacts design and materials requirements, not only in the animal models but also among the (frequently myopic) human patient candidates whom we hope to treat [26]. If the secondary radio frequency coil were to be maintained at a fixed distance from the anterior edge of the hermetic electronics package among the peri-ocular components, e.g., using a fixed-length micro-fabricated connecting structure, a smaller-diameter host eye would force the posterior edge of that electronics package far back into the orbit, thus creating a challenging trade-off for the surgeon during implantation. This design element was optimized through collaboration between our surgical and engineering teams. The micro-fabricated coil support structure shown in Figure 1 was built to accommodate varied host eye shapes by encapsulating the RF coil’s leads in flexible silicone surgical tubing, and by fixing the ends of that tubing segment to the coil support with sutures and to the electronics package via silicone over-molding. These modifications allowed considerable flexibility in the relative placement of the electronics package and the RF coil because of the tubing’s flexibility, while at the same time allowing the surgical field to be less crowded, since the coil assembly could be “flipped over” out of harm’s way until it became time to affix the coil to the sclera.

Turning to considerations for microfabricated intra-ocular components, there are two competing needs. On the one hand, our electrode array must be stiff enough to pass through a flap created in the sclera and an incision in the choroid, which is one of the most vascular tissues in the body. The electrodes must also be able to be advanced to the target sub-retinal location. On the other hand, once the array is advanced to its final resting spot, it is desirable for the array to be flexible, so as to conform to the contour of the sub-retinal space. This segment of the device must be stiff enough to breach an incision in the choroid, yet at the same time, if the distal end of the electrode array retained that same level of stiffness, it might cause long-term biocompatibility issues for the device, including potentially a retinal tear. Our solution to this problem was in part to keep the intra-ocular portion of the flexible electrode array to <15 μm in thickness, and to perforate the electrode array by design using reactive ion etching (RIE) so as to allow fluid exchange through the openings that rest within the sub-retinal space. Further processing details are below. Additionally, to assist in the surgical procedure, we employed a custom-fabricated 75 μm-thick shaped polyimide surgical guide that was laser-cut from a DuPont Kapton® sheet. The distal aspect of the guide was dull (i.e., had rounded edges) but semicircular in shape, which aided implantation of the flexible multi-electrode array through the flap and into the sub-retinal space (see Figure 1). The procedure proceeded by first introducing the stiffer polyimide guide through the cauterized choroidal incision, and then implanting the electrode array by sliding it over and along the guide into the eye, having first raised the retina out of harm’s way by creating a bleb (by injection through a small retinotomy from inside of the eye). The guide was then removed, the electrode array was sutured in place to anchor it to the sclera outside the incision site; the bleb was slowly resorbed. In this manner, a flexible multi-electrode array with or without penetrating posts was deployed through an incision that the array would not otherwise have had enough stiffness to pass through. Further details about the implantation procedure have been recently presented [16]. 

A final mechanical design consideration for a retinal prosthetic’s electrode array and other micro-fabricated implantable components is the selection of encapsulating materials to protect the leads and other structures from ingress of moisture. This consideration also intersects with other factors, such as biocompatibility in the sub-retinal space, and compatibility of the deposition process for the encapsulating material in question with the previously existing component materials that are deposited earlier in the process flow. These intersecting needs may be better understood through examination of Table 2 below. Biocompatibility data are drawn in part from our team’s earlier studies in mini-pig animal models [27] and are supplemented with more recent data from pre-clinical testing of our team’s retinal prosthetic device.

**Figure 2:**
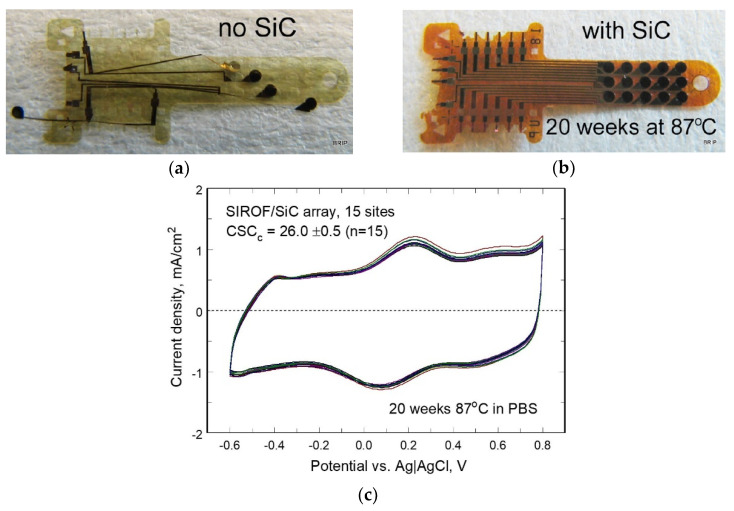
Early micro-fabricated test electrode arrays by the Boston Retinal Implant team. (**a**) The conducting traces interconnecting the contact pads on the left with the 400 μm diameter Iridium Oxide electrodes on the right were encapsulated in HD Microsystems PI-2611 polyimide only, and quickly de-laminated under accelerated soaking conditions in saline solution at 87 °C; (**b**) multi-electrode arrays which were “wrapped” or encapsulated in 0.5 μm thick PECVD a-SiC above and below the conductors (in addition to a polyimide outer coating for scratch protection) survived for over 5 months under the same conditions, which we project to be equivalent to over 10 years at 37 °C; similar bio-stability of SiC has been demonstrated elsewhere [28,29]. (**c**) Cyclic voltammetry curves measured from the multi-electrode array of Figure 2b after 20 weeks of saline soaking at 87 °C demonstrated good, consistent charge storage capacity on all 15 sites.

From the development experiences of earlier generations of our team’s visual prosthesis, we gradually came to appreciate the unique and intersecting challenges that were summarized in Table 2 concerning the impact of added encapsulating films on the bio-stability and biocompatibility of the devices in the eye environment. Early non-hermetically encapsulated visual prosthesis components micro-fabricated by our team had vapor-deposited protective coatings of Parylene-C applied (Figure 1a,b) including the use of organosilane adhesion promoters and careful surface preparation before coating and patterning using oxygen reactive ion etching. Despite these precautions, we found that Parylene-C coatings alone had useful lifetimes in saline environments of at best 1–2 years at 37 °C. This was adequate, for example, for some in vitro and early in vivo testing, but fell short of our ultimate design lifetime target of 10+ years. After evaluating a number of potential encapsulants [27], we eventually settled on “wrapping” the conductors in our arrays with plasma enhanced chemical vapor deposition (PECVD) of amorphous silicon carbide (a-SiC:H) at 325 °C, which showed excellent long-term material and electrical stability in accelerated life tests at 87 °C (see e.g., Figure 2). These inorganic encapsulating layers were used above and below each layer of conductors, which we kept near to the neutral axis of the arrays to minimize any effects of internal film stress. The presence of a-SiC:H layers in the multi-layered electrode array structure also contributed to their overall stiffness, which was compensated for in part by adjusting the thickness of the outer polymer layers of the micro-fabricated components for scratch protection. 

While our combined inorganic and organic encapsulation strategy was evaluated through numerous tests, a systematic study of the effects of coating thickness (and thus stiffness) on long-term device lifetime was not performed from first principles. Rather, in the case of inorganic encapsulation layers deposited e.g., by plasma enhanced CVD, their natural stiffness was so much greater than that of the flexible polyimide substrates that we had microfabricated that we limited the thickness of such layers to only that which was necessary to have reasonable confidence of pinhole-free coatings. In the case of the a-SiC:H coatings used for the test devices of Figure 2, this thickness was 0.5 μm, but later process optimization led to the reduction of the required SiC layer thickness to 0.1 μm for top or bottom layers, and 0.25 μm for any middle layers. Other materials that we investigated in Table 2 either lacked sufficient biocompatibilty, biostability, or both.

Another matter relating to mechanical properties of our implantable multi-electrode array components in particular was the channel count needed in order to be able to stimulate sufficient numbers of sites to have a reasonable likelihood of restoring “useful” vision. We estimated for our third-generation retinal prosthesis that at least 256 channels would be required, based on the findings of a number of teams in the field over the last 20+ years that have recently been reviewed [3,4]. Since no de-multiplexer was included in our system in a distal region near the stimulation sites beneath the retina, this meant that >256 individual traces or leads would need to be incorporated along the length of the array from the stimulator package containing the circuitry, to the electrode locations within the eye. A quick calculation revealed that even for a modest center-to-center spacing of adjacent conducting traces in the leads of 20 μm, a single micro-fabricated layer containing side-by-side leads to each electrode would need to be >5 mm wide. Not only would a multi-electrode array of this width likely not conform well to the approximately spherical contour of the eye, but it would also require a relatively large incision to access the sub-retinal space; broadly speaking, our surgical experience has shown that reliable insertion into the sub-retinal space can be achieved using electrode arrays that are <5 mm wide that are implanted through sclera flaps that are ~6 mm in width [16]. The pitch between electrode sites at the distal end of the array, then, was determined largely due to surgical factors. Smaller- or larger-pitch arrays would have been feasible to micro-fabricate, but may not have been as feasible to implant. “Pitch” between electrode sites here refers to the nearest-neighbor distance in a nominal hexagonally close packed configuration, which was used to save space. In contrast, the rigid, disc-shaped hermetic package containing our stimulating electronics was limited in size as well, since a larger package would likewise have been difficult to implant behind the eye in the orbit. The pitch between signal feedthroughs in that package in turn determined the overall package’s 11.5 mm diameter. In that case, it was the limitations of the fabrication process for creating high temperature co-fired ceramic parts that determined the spacing. While it is true that high channel counts can and have been achieved, e.g., by the use of alternative, photodiode-based visual prosthesis designs, those approaches are entirely different, make use of the capabilities of silicon foundries used to create such devices, and have been reviewed elsewhere [3,4].

**Figure 3:**
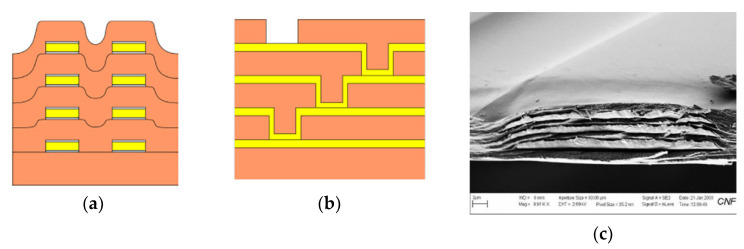
(**a**,**b**) Schematic cross section of a four-layer, flexible microfabricated lead structure for a multi-electrode array in which the separate, insulated 0.05 μm Ti/1.5 μm Au/0.05 μm Ti metal traces (light color) are separated by HD Microsystems PI-2611 polyimide layers (not to scale) and interconnected with metalized via holes; (**c**) SEM micrograph of a cross section of an as-fabricated structure of this type; as the number of metal layers increased, step coverage over previously patterned traces with photoresist became increasingly difficult, to the point where the use of >4 such layers introduced too many process defects [30]. Multiple layers permit the width of a high-density multi-electrode array to be reduced, and also permit crossing signals over from one layer to another.

Given the channel count desired for our design, then, it was concluded that for a high-density retinal prosthetic, the large number of independent sites involved required that the leads be printed on multiple layers of metal. Each layer of conductors required inorganic encapsulation above and below the metal traces to insulate them from one another, which also increased the overall required silicon carbide thickness and thus, the stiffness of the multi-electrode arrays. We also studied the feasibility of increasing the number of layers of conducting traces to as many as 4, which would be required in the case of very high channel counts unless a distributed system involving the use of signal multiplexing and de-multiplexing were employed. In Figure 3, test structures and cross sections of sample four-layer devices that were fabricated are shown [30]. The electrode arrays shown in Figure 1, however, were fabricated with just two layers of conductors, again using vias between the layers; this process is outlined in Table 3. Using two layers of traces, we were able to accommodate twice the channel count in the same electrode array width, and we were also able to “cross over” or under a trace on an adjacent layer by creating conductive vias between the layers on either side of the area that was desired to pass beneath (or over).

The final issue concerning mechanical properties of the implanted components concerned the fit of the as-fabricated 2-D micro-fabricated components to the real, 3-D features of the eye. This was a particularly challenging issue on several fronts. First, there are natural variations in anatomy of the eye including its length (which correlates with the natural refractive state of the eye) and the insertion sites of the extra-ocular muscles, which differ between porcine and human subjects.

**Figure 4:**
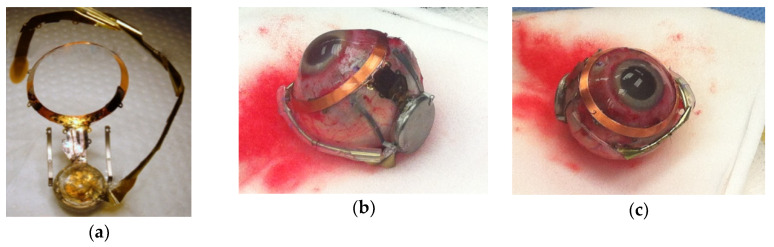
Photographs of the implanted components of our prosthesis. (**a**) A previous-generation coiled-cable electrode array that accommodated a variable distance between the implanted neuro-stimulator package and the insertion site into the sub-retinal space. A flexible, 12 μm-thick array is helically wound around a medical grade silicone tube; (**b**,**c**) The same generation device is shown implanted around a human cadaver eye after its enucleation. Note, the eye shrinks in size *post mortem*, thus the prosthesis fit in this example is not exemplary of what the appearance of the fit would be for a living subject that had the same ocular size pre-operatively.

Specifically, we sought a design that could accommodate variations in our animal model (needed for pre-clinical testing) that also would be suitable for human eyes that also have variations. And, we wished to build electrodes that would be usable across a range of eye sizes without having to customize arrays for individual patients. Thus, a degree of adjustability was desirable in our design. After much trial-and-error over the course of a number of early surgical trials, we developed two array designs that were workable. A first design evolved which employed a medical grade silicone tube as a physical support for a helically wound coiled-cable type lead, which was inspired in part by flexible, implantable helix type wire-based leads that are in common use in other medical devices. The use of the coiled arrays required a much longer physical length of the arrays, because much of the length was consumed in the coiling process but the coiled arrays were easily re-positioned without significant creasing or sharp bending of the coiled structures. And, the coiled assemblies could easily be passed beneath the eye muscles by simply elevating them with a muscle hook and passing the array beneath with the free hand. An example of an inactive coiled-cable array mockup fitted around a human cadaver eye can be seen in Figure 4. 

Ultimately, our ~4 mm wide, two-dimensional implementation of a 2-layered multi-electrode array was refined through pre-clinical testing. This required dealing with a fundamental problem that micro-fabricated structures for medical applications often encounter, and that is particularly true for organs such as the eye. The micro-fabricated structures are frequently created on planar surfaces, such as silicon wafers, for ease of handling and processing. However, the host tissue, i.e., the mini-pig eye in the case of our pre-clinical tests, had a complex 3-D shape and a diameter of ~23 ± 2 mm. The issues that were related to laying our fabricated 2-D structures down onto 3-D surfaces can easily be visualized by wrapping a strip of paper around a ball. There is a natural tendency for the 2-D object, such as the paper in this example, to buckle or to protrude in some manner when portions of 2-D objects are affixed to the 3-D surface, and the edges of the very thin 2-D parts, such as the flexible electrode arrays here, can be sharp. This deformation with a sharp leading edge can lead to erosion of the conjunctiva when thin, flat prosthesis components are implanted beneath it, which we in fact initially did experience [25]. To facilitate a degree of adjustability to different host eye sizes, and to allow the 2-D multi-electrode arrays the ability to lay flat on the sclera surface, we eventually adopted a serpentine shape for the arrays, which can be seen in Figure 1. Even these structures, which lay relatively flat on the eye surface, required segmentation of the two-layered multi-lead structure into strips or groupings of 10 μm wide metal traces that were interconnected by thin polyimide “bridges.” In this way, the natural tendency to buckle was minimized, since a segmented structure lays flatter than one which crowds all of its leads into a single group. We also incorporated suture loops along the perimeter of the electrode arrays’ length to facilitate the attachment of the arrays at key stress points—again, in the service of fixing the arrays in position with minimal protrusion from the sclera surface. Further, we found that the order of the surgical procedure for implanting a device in the orbit was also important. For example, it is possible to prevent the need to compress, bunch the arrays, or stretch the arrays to span the gap between the sclera incision site and the location of the packaged electronics, by first marking on the surface of the eye the desired location of the insertion site for the electrode array, which then facilitates selecting a compatible location for the hermetic package to which it is attached. 

### 2.2. Process Flows for Microfabrication

Our multi-electrode array fabrication processes needed to consider the positioning of the distal retina-facing side of the array, in relation to the contact pads on the proximal side of the micro-fabricated structure that connect with the signal feedthroughs in the hermetic electronics package. This decision had significant manufacturing ramifications. A sub-retinal electrode array fabricated with the contact pads for thermosonic bonding to feedthroughs on the same side of the array as the iridium oxide stimulating electrodes required that the package’s feedthrough disc face away from the eye surface. Consequently, the multi-electrode array’s flexible leads exit the package at some elevation (in our case, about 2.5 mm) above the sclera surface, and the silicone strain relief that was designed for that joint needed to take that into account. The volume of the elevated electronics case and the attached, micro-fabricated leads necessitated placement away from the delicate conjunctival tissue in the front of the eye and around the back of the eye where these structures could be easily accommodated by the compliant fatty tissue of the orbit. Even with this posterior placement, the relative bulk of these structures produced some tension, which we buttressed by placing the anterior edge below the sturdier Tenon’s capsule. 

**Figure 5:**
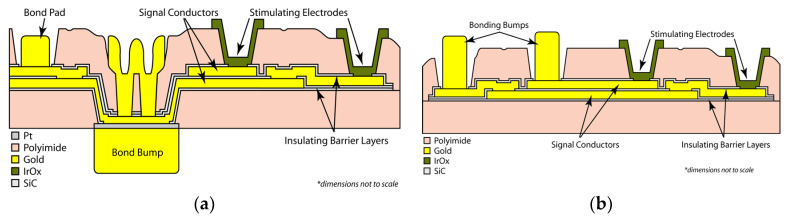
Two process flows for microfabrication of flexible, implantable planar multi-electrode arrays for use in retinal prostheses. (**a**) A double-sided process, in which the contact pads may be located on either or both sides of the array; (**b**) A single-sided process, in which the contact pads for bonding to the stimulator package are on the same side of the array as the iridium oxide stimulating electrodes themselves (see also Table 3).

Thus, a simplification of the micro-fabrication complexity (by fabricating multi-electrode arrays with features patterned on one side only) needed to be “traded off” with a slightly more nuanced step in the surgery. Our team also tried a different micro-fabrication approach that placed the electrodes on the reverse side of the array from the pads that connect to the stimulator package, but this substantially increased the difficulty and number of process steps required. This modification is illustrated below in Figure 5; both single- and double-sided multi-electrode arrays were fabricated, but our current-generation retinal prosthesis uses the simplified fabrication sequence outlined in Table 3 and in Figure 5b.

The process flows of Figure 5 for single- or double-sided fabrication of electrodes had certain critical properties in common which were important for successful device fabrication. First, it was essential that the flexible components could be nondestructively removed from their host wafers once all process steps were completed. This required that the base layer(s) of the structures cling sufficiently to the host wafer to allow for reliable alignment of the mask layers to one another and for maintenance of planarity during processing. At the same time, we wanted to be able to peel the completed parts from the surface of the host wafer at will. This required careful attention to the moisture levels on the host silicon wafers prior to the application of the first layer of HD Microsystems PI-2611 polyimide, taking care not to unduly expose the wafers to water for extended periods during the fabrication sequence. In this way, the natural, gradual loss of adhesion of polyimide to Si wafers upon soaking in DI H_2_O can be used to advantage; soaking for 24 h was usually sufficient to loosen the completed/singulated devices from their host substrates.

As mentioned above, another feature of the planar electrode manufacturing process that was common to either single- or double-sided arrays was the need to “wrap” each conducting trace on the multiple layers with an inorganic encapsulant, for example amorphous SiC:H in this case. Polyimide or Parylene-C polymers alone were not sufficient to electrically insulate the traces from one another throughout long-term in vitro accelerated lifetime tests, and moreover, we found that such structures would blister and de-laminate over time in saline environments despite careful cleaning before coating. The use of an inorganic primary encapsulant, however, has enabled our devices to survive for a projected lifetime of over 10 years at 37 °C (see e.g., Figure 2); the outer polymeric encapsulants primarily serve as scratch protection, improve the ability to handle the arrays in surgery, and also perform the release function from the host wafer.

The evaporated metallization that we patterned using a standard photoresist lift-off method likewise served multiple functions in the microfabrication process (see Table 3). The need to deliver current to the electrodes with negligible series resistance due to the leads was primarily addressed by using thermally evaporated gold that comprised the bulk of the trace volume. The end-to-end resistance of the 0.425 μm thick traces was <500 Ohms, which was deemed acceptable, since the electrode-tissue interface impedance for retinal prostheses was generally an order of magnitude higher, and the in vivo impedance was found to be even greater, often in the range of 10–20 kOhms. On the top and bottom of each metallization stack was a Titanium adhesion layer of 0.025 μm thickness. It was found when curing the upper polyimide layers at 350 °C in a N_2_ ambient that the Titanium and Gold formed an inter-layer which altered the appearance of the traces and could also give rise to abnormally high impedances. In part to prevent this phenomenon, and in part to prevent accidental exposure of the Gold metallization in the SF_6_/O_2_ reactive ion etching process during etching of the overlying a-SiC:H layer, a thin 0.05 μm-thick film of Platinum was added by electron beam evaporation between the Gold and the Titanium layers on the upper side.

The sputtered iridium oxide (SIROF) electrode material was deposited by reactive sputtering of an Iridium metal target in the presence of moist N_2_ carrier gas; process details have been summarized elsewhere [31]. The nature of sputtered films is that they arrive at the substrate in an isotropic manner, which is excellent for providing uniform coverage but can sometimes present a challenge for conventional patterning techniques such as lift-off. In practice, the SIROF material did lift off readily using ordinary photoresist processing methods, but the sidewalls of the SIROF-coated electrodes would at times separate from the portion of the electrode that was deposited in the open areas that had been patterned to receive the film—particularly after a period of hydration and repetitive pulsing. While this altered the effective electrode geometric surface area somewhat, there appeared to be no deleterious effects in vitro or in vivo. We also noted that the as-fabricated SIROF films did not have the full charge storage capacity that they are capable of immediately after fabrication; rather, a break-in period significantly improved the charge capacity of both planar and penetrating SIROF-coated electrodes, and this is discussed further below.

**Figure 6:**
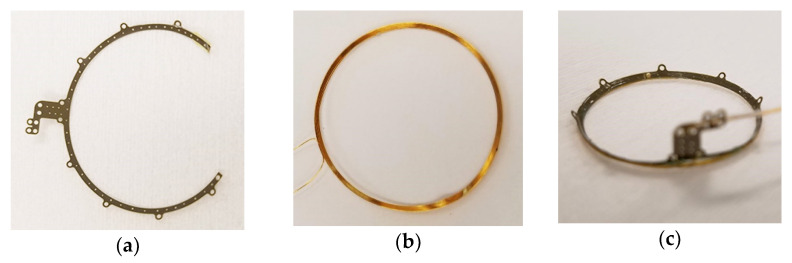
Microfabricated supporting structure for supporting and suturing a 19 mm diameter wire wound secondary radio frequency coil around the cornea (see also Figure 1). (**a**) A base layer of HD Microsystems PI-2611 polyimide was applied to a host Si wafer, electroplated Au was added to mechanically reinforce the structure, and the support perimeter and suture holes were etched by O_2_ RIE; (**b**) An Au wire coil wound on a spherical form so that it would lay flat on the sclera surface; (**c**) The assembled coil support with an incoming silicone tube to protect the coil wires from handling and to allow flexibility of coil placement relative to the electronics package.

The process for creating the supports for the wire-wound gold radio frequency coils differed substantially from that for fabricating the multi-electrode arrays above, but retained certain features in common. For example, the same method for releasing the completed coil supports from the host substrate was used, with a base layer of HD Microsystems PI-2611 polyimide. In the case of the coil supports, their primary function that they served was mechanical and not electrical, in that the coil wires terminated on connection pads prepared for that purpose on the proximal end of the electrode arrays, and no conductors were used to carry current in or through the coil supports themselves. Rather, electroplated Au metallization was instead used for its ability to strengthen the suture holes that were etched around the perimeter for affixing the coil to the sclera. Once the coil supports were freed from the host wafer, the wire-wound coils were attached to them using Kwik-cast, a medical grade silicone adhesive. Completed coil supports are shown in Figure 6.

**Figure 7:**
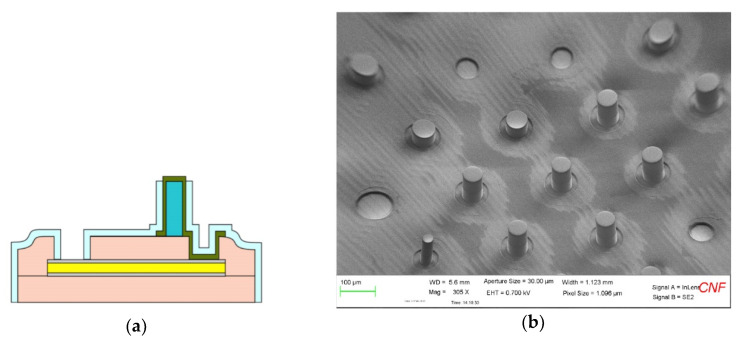
(**a**) A cross section diagram of a flexible, microfabricated electrode array with a penetrating post formed from photo-imageable SU-8 epoxy (in dark blue) whose sidewall is insulated with Parylene-C (light blue). The fabrication process is similar to the planar electrode steps of Table 3, except that the SU-8 films are patterned prior to the SIROF deposition and liftoff, and Parylene-C coating; (**b**) A SEM micrograph of a completed, hybrid penetrating- and planar-electrode array, containing posts that are either 50 or 100 μm-tall (two separate 50 μm layers of SU-8 were applied).

Most recently, our team has made advances in microfabricating and implanting subretinal multi-electrode arrays that contain both planar electrodes, and electrodes atop penetrating posts for stimulation of the inner retinal layers, each of which are SIROF-coated; these have been well tolerated in a number of animal trials [16]. The process for creating “hybrid” penetrating and planar electrode arrays differed from the basic planar multi-electrode array process in that prior to sputtering the SIROF electrode material (but after the exposure of the metal underlying the electrode sites), SU-8 photo-imageable epoxy was applied to the entire wafer surface and patterned; special care needed to be taken in the extended soft baking step. On the one hand, this prevented adhesion between the photomask with the desired post pattern and the coated wafer surface, and on the other hand, the soft baking process improved the adhesion of the posts themselves to the host surface after developing the pattern. A SIROF film was sputtered onto the passive penetrating posts, creating a conducting path from the base of the posts to the tips; the 50 μm and 100 μm-tall posts had a top-to-bottom impedance of <40 Ω, which introduced negligible additional series resistance to the sites. After the SIROF patterning, the arrays were coated in 1 μm of Parylene-C by vapor deposition, and the tops of the posts were exposed by reactive ion etching in an O_2_ plasma. Special care was needed to properly protect the posts and the surrounding electrodes during this etching step with photoresist, and applying the resist using a spraying technique with a Suss Microtec Gamma Cluster Tool with a spray coating module provided this protection. A schematic cross section diagram of a multi-electrode array with an incorporated penetrating post and a micrograph of a completed hybrid array with mixed planar- and penetrating electrode sizes is in Figure 7.

## 3. Results

The Boston Retinal Implant team has created three generations of retinal prostheses with microfabricated components since its inception. Our team was the first to employ flexible polyimide-based epi-retinal electrode arrays for early acute clinical testing in blind patients [1,2]. The psychophysical outcomes from that acute study in blind volunteers, which were obtained using multi-electrode arrays constructed without the inorganic encapsulating layers that were later found to be essential to long-term biostability, nevertheless encouraged our team to create our first 16-channel wirelessly powered, chronically implantable stimulators that had many of the attributes of our current devices (see Figure 1 and [25] for example). 

**Figure 8:**
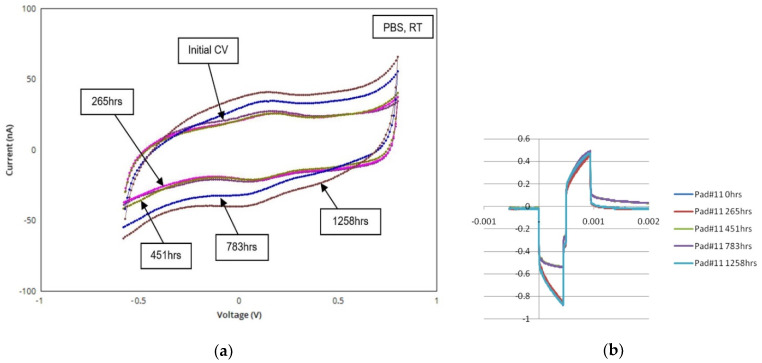

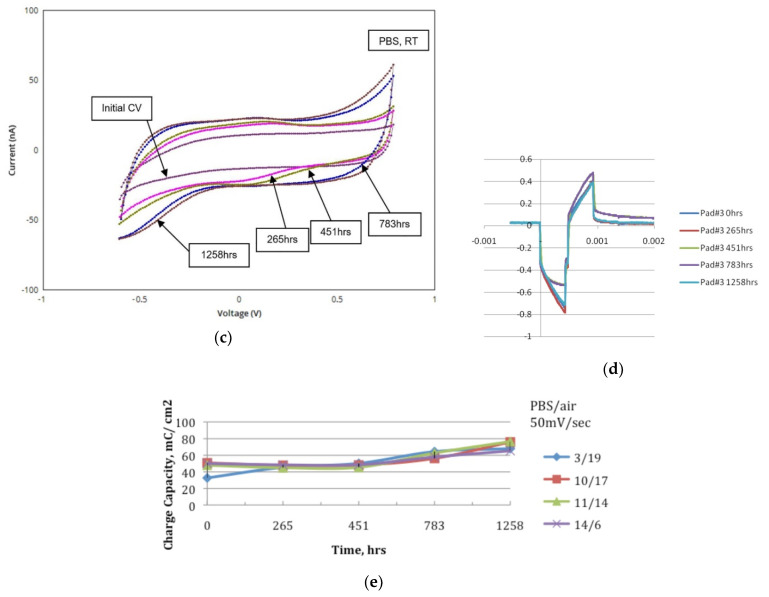
(**a**,**b**) Cyclic voltammograms versus time for a 40 μm diameter microfabricated SIROF-coated penetrating post electrode. Note the evolution of the charge capacity, proportional to the enclosed area; the corresponding voltage waveforms for 1 msec bi-phasic current pulses are shown at right. (**c**,**d**) CV plots and voltage waveforms over time for a corresponding 40 μm diameter planar electrode, respectively; (**e**) charge capacity versus time for a 40 μm diameter planar electrode (#3/19; blue trace) and for three, 40 μm diameter penetrating post electrodes (#’s 10/17. 11/14. and 14/6; red, green, and purple traces) showing gradual improvement over 1000+ hrs. of pulsing, likely due to hydration of the SIROF films.

As mentioned, the primary components in retinal prostheses that require microfabrication technology are the high-density multi-electrode arrays, and/or the structures that form the neural interfaces. These devices ideally have a high charge capacity, owing to the current densities required to achieve useful phosphenes—especially for small electrodes. This led our team to employ Iridium Oxide electrode coatings early in our device development because of the margin of stimulation safety they provide. The deposition process for IrOx and early electrode characterization has been presented elsewhere [31], but Figure 8 shows cyclic voltammograms and voltage waveforms from identical area planar- and penetrating-post electrodes, such as on the “hybrid” multi-electrode arrays shown in Figure 7. 

The expansion of the charge capacity and enclosed area in the voltammograms are typical of SIROF electrodes with repeated cycling in saline solution, and this outcome was similar for both planar- and penetrating-electrodes. The changes to the electrode voltage waveforms upon repeated bi-phasic current pulsing can also be seen. The differences are attributed to hydration of the SIROF films and expansion of the effective surface area of the sites from the films’ as-deposited state. This feature is best taken into account when “burning in” a new electrode array on the bench top prior to implantation, for example. Platinum electrodes deposited by conventional physical vapor deposition processes, which we fabricated in the early years of our team’s device evolution, did not require such treatment. SIROF charge capacity was also found to continue to increase even after more than 1000 hrs of continuous pulsing, indicating that the beneficial effect of exercising the electrodes in saline environments continues beyond an initial break-in period.

Our planar- and penetrating-electrode arrays have also been successfully implanted sub-retinally in Yucatan mini-pig models as part of a number of pre-clinical biocompatibility tests by our colleagues in Boston and also at the University of Louisville, KY USA; the procedure for sub-retinal multi-electrode array implantation is described in greater detail elsewhere [18]. The key results are that with proper layout and attention to the microfabrication details outlined above, such as a serpentine design that can accommodate variations in eye size/electronics module placement and combined inorganic/organic encapsulation schemes, electrodes can deliver bi-phasic current pulses to the target tissue over weeks and months post-operatively, with only modest, localized tissue responses. This was true even for penetrating post electrodes, which caused minimal gliotic response in the host retina; the devices could also be explanted without significant eye trauma [16,17]. 

## 4. Discussion

Microfabrication technology for retinal prostheses, while not as mature as processes for integrated circuit fabrication, has nevertheless progressed in important ways to fill the design input requirements for visual prosthetics. In particular, our team has evolved segmented, serpentine high-density multi-electrode arrays with >256 electrodes for sub-retinal stimulation, having excellent SIROF charge capacity and demonstrated biostability. These SiC-encapsulated electrodes have leveraged virtually all of the technologies that are incorporated in current clean rooms for semiconductor fabrication, including photolithography, physical vapor deposition, and chemical and reactive ion etching, to name a few. While silicon wafers are used as host substrates, the microfabricated components are removed from their host surfaces upon completion; precision lithography, which is necessary to create high-density arrays, would not have been possible without the Si starting material. 

Visual prostheses will likely remain technology drivers for implantable medical devices for the foreseeable future. The high channel counts required to create complex patterns of phosphenes that correspond to images that are captured in the user’s environment will likely drive the fabrication of even greater density visual prosthesis components in the future—if the packaging of the implantable electronics can keep pace. This is especially true as visual prostheses increasingly focus on treatment not only of severely blind patients, but also of individuals with age-related macular degeneration who retain some useful peripheral vision but require restoration of higher-acuity central visual function. Future work, in fact, will likely lead to the manufacture of integrated medical devices for vision restoration that incorporate not just micro-fabricated electrodes, but also stimulation and recording electronics and fully integrated leads and chip scale packaging; such implantable prostheses are indeed already under development.

## 5. Patents

Patent applications have been filed for certain of the fabrication methods presented here, but they are still pending at the time of article submission.

## Figures and Tables

**Table 1:** Some common micro-fabrication challenges unique to retinal prosthetics, and methods used by the Bionic Eye team to address these challenges.

Microfabrication Related Challenge	Brief Summary of Methods Used
Adequate stiffness of flexible electrode array enabling insertion through an incision in the choroid without damaging any penetrating structures that support stimulation sites	Use of silicon carbide “wrapping” to encapsulate individual conducting traces, and careful SU-8 epoxy processing to form strong supporting posts for penetrating electrodes, if present
Resistance to damage from abrasion of extra-ocular components due to surgical handling and subsequent, normal eye movements	Use of polyimide and Parylene-C outer coatings, and “coil supports” beneath implanted secondary radio-frequency coils for power and data transfer
Ability of fabricated components to conform to different shapes and sizes of host eyes	Use of grouped conducting traces in electrode arrays that are joined by polyimide “bridges,” and a meandering 2-dimensional layout designed to wrap neatly around a 3-dimensional eye
Need for high channel count without increasing the width of the electrode array to the point where it becomes difficult to implant, or buckles when bent	Use of multiple layers of metal traces and electrodes, separated by silicon carbide dielectric layers, with vias to transition signals from layer to layer

**Table 2:** Comparison of properties of potential encapsulating materials for micro-fabricated, implantable retinal prosthesis components to improve long-term biostability.

Encapsulating Material	Mechanical Properties	Biocompatibility in Sub-Retinal Space	Micro-Fabrication Process Compatibility	Effectiveness in Improving Bio-Stability
Parylene-C	Pliable	Good	Good	Poor ^1^
Al_2_O_3_	Brittle	Poor	Average	Poor ^2^
SiN_X_	Strong, somewhat Brittle	Average	Good	Average ^3^
a-SiC:H	Strong, somewhat Brittle	Good	Good	Excellent ^4^

^1^ Lasted 1–2 years @ 37 °C, see e.g., [25]. ^2^ Retina tissue responded adversely [27]. ^3^ Projected to last 5+ years @ 37 °C in accelerated saline soaking tests. ^4^ Projected to last 10+ years @ 37 °C, see e.g., [28,29].

**Table 3:** Schematic microfabrication process flow summary for a flexible multielectrode array with Iridium oxide stimulating sites and two layers of metallization for signal routing. Features are not drawn to scale, for visibility.

Process Step Summary	Layer-by-layer Schematic Representation (Cross Section)
Spin coat and cure 7 μm base layer of HD Microsystems PI-2611 polyimide	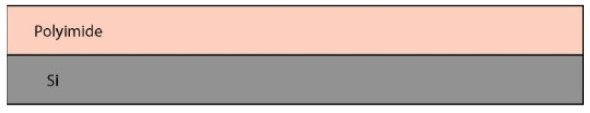
Deposit lower 0.1 μm layer of a-SiC:H by plasma enhanced chemical vapor deposition (PECVD) and liftoff 0.025 μm Ti/0.3 μm Au/0.05 μm Pt/0.025 μm Ti layers	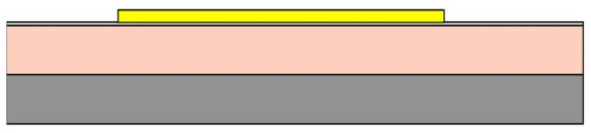
Deposit 0.25 μm a-SiC:H interlayer and pattern vias by reactive ion etching	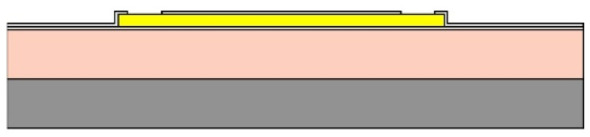
Deposit and liftoff second conductor layer identical to the first one	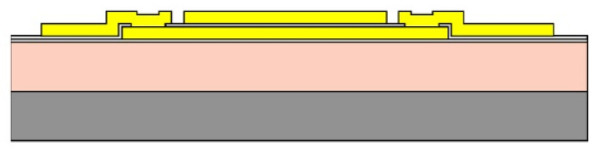
Deposit and top layer of PECVD a-SiC:H	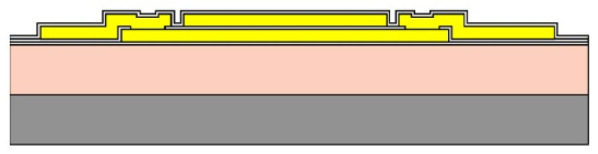
Reactive ion etching of patterned a-SiC:H in SF_6_/O_2_ plasma by reactive ion etching (RIE)	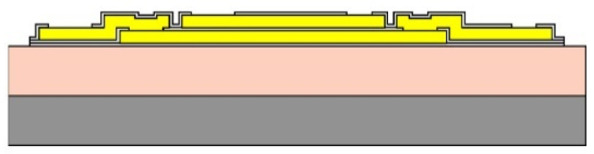
Spin coat and cure 4.5 μm top layer of HD Microsystems PI-2611 polyimide, and pattern openings by O_2_ RIE	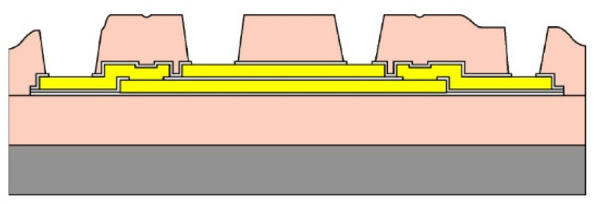
Pattern and liftoff 0.04 μm Ti and 0.2 μm sputtered iridium oxide (SIROF) on electrode sites	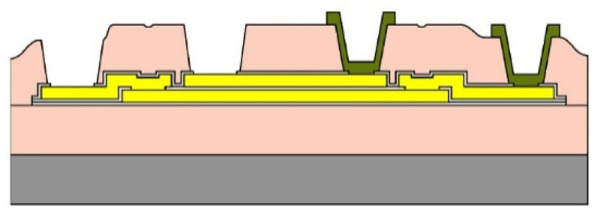
Pattern and etch polyimide perimeter of multi-electrode arrays by O_2_ RIE	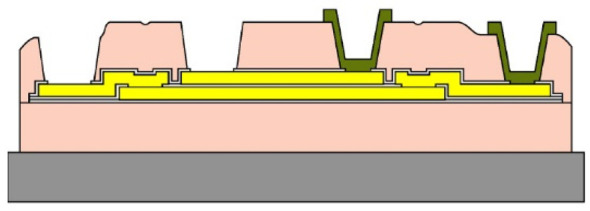
Pattern and electroplate Au bumps for thermosonic bonding to package feedthroughs; soak to remove 12 μm thick arrays from Si carrier in DI H_2_O